# Personal News Items

**Published:** 1915-08

**Authors:** 


### Personal News Items.

Dr. and Mrs. W. N. Young are spending a month visiting in Boston and New York.

Dr. E. C. Durham of Hico has gone to Chicago to study in the Chicago Eye, Ear, Nose, and Throat Hospital.

Dr. E. O. Callaway of Bowie has gone to Cleveland, Ohio, where he is now one of the internes in the city hospital.

Dr. George Cyrus Kindley of Galveston spent the month of July at Rochester, Minn., studying under Mayo brothers.

Dr. Bruce of Nacogdoches has gone to Colorado for a vacation. He will return to his home about the middle of August.

Dr. Margaret Holliday of Austin has gone for a six weeks' trip to Yellowstone National Park and the two expositions in California.

Dr. C. D. Francklow of Shiro has passed the examination taken before the State Board of Medical Examiners and will practice in Shiro.

Dr. T. N. Roach of Gilmer, Texas, and Dr. J. W. Mickle of Memphis, Texas, have formed a partnership and will practice in Memphis.

Dr. John H. Sewell of Johns Hopkins was in Jacksboro visiting relatives. Dr. Sewell will later form a partnership with Beall & Beall of Fort Worth.

Dr. Shook has returned from Chicago, where he took a special lecture course. He also attended the national tuberculosis conference for several days at Detroit.

Dr. and Mrs. Hugh White of El Paso are spending a few weeks in Virginia.

Dr. Geo. W. Reagan of Groesbeck has passed the examinations held by the State Board of Medical Examiners. He will probably practice medicine in Groesbeck.

Dr. and Mrs. William Gammon and little son have gone to Schenectady, N. Y., to visit Mrs. Gammon's mother, Mrs. S. Stern. Dr. Gammon is a citizen of Galveston.

Dr. William H. Welch, pathologist of Johns Hopkins University, has gone to China, where he will take part in the work of systematizing medical education of the Orient.

The following doctors have visited California this summer: Dr. G. T. Vineyard, Amarillo; Dr. A. P. Lacey, Mt. Enterprise; Dr. A. S. McDaniel, San Antonio; Dr. F. R. Adamson, Anson; Dr. I. B. Nofsinger, Elgin; Dr. S. C. Relyea, Ladonia; John S. Turner, Dallas; Dr. J. P. Oldham, San Antonio; Dr. J. W. Tottenham, Hempstead.

Dr. W. D. Cross, secretary of Navarro County Medical Society, read a paper at Corsicana recently, in which he claimed that there were more than five hundred cases of pellagra in Navarro county and that the disease was spreading at an alarming rate. This paper embodies a report from physicians all over the county. It is interesting and instructive.

